# Correction

**DOI:** 10.1080/13880209.2023.2210885

**Published:** 2023-05-08

**Article title:** Sanguinarine inhibits melanoma invasion and migration by targeting the FAK/PI3K/AKT/mTOR signalling pathway

**Authors:** Qi, X., Chen, Y., Liu, S., Liu, L., Yu, Z., Yin, L., Fu, L., Deng, M., Liang, S., & Lü, M.

**Journal:** *Pharmaceutical Biology*

**Bibliometrics:** Volume 61, Number 01, pages 696–709

**DOI:** https://doi.org/10.1080/13880209.2023.2200787

There were the below errors when this article first published online.

1. In Figure 1(F), the pictures of A375 cells at 1.5 μM and 2 μM concentrations were repeated.
2. In Figure 5(A), the images of A375 cells in the Control group at 0 h and at 1 μM concentration were repeated.
3. In Figure 6(D), the images of A375 and A2058 cells at 1.5 μM and 2 μM concentrations were repeated.
4. In Figure 9, the images of the Heart group at 2 mg/kg and 4 mg/ kg concentrations were repeated.

Figures 1, 5, 6, and 9 are now corrected and the article has been republished online.

